# Pathogen burden and leukocyte telomere length in the United States

**DOI:** 10.1186/s12979-020-00206-9

**Published:** 2020-11-19

**Authors:** Grace A. Noppert, Lydia Feinstein, Jennifer B. Dowd, Rebecca C. Stebbins, Emma Zang, Belinda L. Needham, Helen C. S. Meier, Amanda Simanek, Allison E. Aiello

**Affiliations:** 1 Social Environment and Health, Survey Research Center, Institute for Social Research, University of Michigan, Ann Arbor, MI USA; 2 Carolina Population Center, University of North Carolina at Chapel Hill, 123 West Franklin St., Chapel Hill, NC 27516 USA; 3 Department of Epidemiology, Gillings School of Global Public, University of North Carolina at Chapel Hill, 123 West Franklin St., Chapel Hill, NC 27516 USA; 4 Social & Scientific Systems, Durham, NC USA; 5 Leverhulme Centre for Demographic Science, Department of Sociology, University of Oxford, Oxford, UK; 6 Department of Sociology, Yale University, New Haven, CT USA; 7 University of Michigan School of Public Health, Ann Arbor, MI USA; 8 Joseph J. Zilber School of Public Health, University of Wisconsin-Milwaukee, Milwaukee, WI USA

**Keywords:** Telomere length, Biological aging, Persistent infections, Geroscience, Immunosenescence

## Abstract

**Background:**

Prior studies in humans have suggested that telomere shortening may be accelerated by infection, but research on multiple pathogens and use of large population-based study samples has been limited. We estimated cross-sectional associations between seropositivity to five persistent pathogens (Herpes Simplex Virus Type-1 (HSV-1), Herpes Simplex Virus Type-2 (HSV-2), cytomegalovirus (CMV), *Helicobacter pylori* (*H.pylori*), and Hepatitis B) as well as total pathogen burden and leukocyte telomere length. Data were derived from the National Health and Nutrition Examination Survey (1999–2000) for individuals 20–49 years of age, *N* = 1708. We analyzed the influence of each pathogen separately, a pathogen count score and a latent class model of pathogen burden on log telomere length using linear regression models, adjusted for covariates.

**Results:**

Individuals in a latent pathogen burden class characterized by high probabilities of infection with HSV-1, CMV, and *H. pylori,* had significantly decreased log telomere length (− 0.30 [95% CI: − 0.36, − 0.24]) compared to those in a latent class characterized by low probabilities of all five infections. There were limited significant associations using other pathogen measures.

**Conclusions:**

These results suggest that infection with specific combinations of pathogens may be one mechanism contributing to accelerated cellular senescence with possible origins early in the life course.

**Supplementary Information:**

The online version contains supplementary material available at 10.1186/s12979-020-00206-9.

## Background

Telomeres are the repeated nucleotide sequences that cap the end of chromosomes to protect them from deteriorating or fusing with other chromosomes. Although telomere shortening is influenced by normal chronological aging [1], accelerated shortening is an indicator of accelerated cellular senescence [2] and has been linked to a growing number of adverse health conditions, including cancer [3], cardiovascular disease [4, 5], inflammation [6], and mental illness [7], as well as a decreased immune response to vaccines [8]. Some studies have also shown an association between shorter telomere length and increased mortality rates [9, 10]. While age is a strong determinant of telomere length and attrition, other determinants such as genetics, socioeconomic status, and health behaviors (i.e. diet, exercise, and sleep) have also been identified [11–13]. For example, there is an observed genetic component to telomere length with evidence from twin studies suggesting nearly 70% of individual variability is explained by genetics [14]. Results from studies examining the role of socially patterned exposures, such as education level, and telomere length have been mixed; while some studies find that individuals with less than a high school education have significantly shorter telomeres than their more educated counterparts [15], others report no associations between education and telomere length [16, 17]. Accordingly, the mechanisms underlying telomere length variability between individuals are unknown. Further clarification of the socioenvironmental factors that result in variability in telomere shortening and explanation of the differences between individuals’ telomere length is needed to improve our understanding of telomere biology.

Recent studies in humans have suggested that telomere attrition may be accelerated by infection with specific pathogens, including cytomegalovirus (CMV) [13, 18], Epstein-Barr virus [19], human immunodeficiency virus (HIV) [20], hepatitis C virus [21], malaria [22] and *Helicobacter pylori* [23]. These observational data are also supported by animal studies, which have experimentally demonstrated a causal effect of infection on accelerated telomere attrition [24–26]. Chronic inflammatory responses are hypothesized to occur secondary to a number of different pathogen exposures, indicating that infection with a higher number of pathogens may have a cumulative effect on inflammation [27]. In addition, it may be that persistent infections also increase the presence of reactive oxygen species, as well as induce autoimmune changes both of which could contribute to accelerated cellular aging [28], including declines in telomerase activity and telomere length.

While the majority of prior studies have implicated individual pathogens in the etiology of telomere shortening, it has been hypothesized that total pathogen burden may also influence telomere biology [29]. In two recent studies conducted by the authors in two different populations, total pathogen burden was statistically significantly associated with shorter telomere length [30, 31]. The study by Aiello et al. showed that increased pathogen burden, including infection with CMV, herpes simplex virus (HSV), *Helicobacter pylori* (*H. pylori*) and *Chlamydia pneumoniae,* was associated with significantly shorter telomere length in females but not males participating in the US-based Multiethnic Study of Atherosclerosis (MESA) [30]. Next, a study by Dowd et al. showed that seropositivity for four or more herpesvirus infections (CMV, HSV-1, human herpesvirus type 6, and Epstein- Barr virus) was significantly associated with shorter telomere length over time among males and females participating in the UK Whitehall study [31]. While these studies provide strong evidence of an association between total pathogen burden and telomere shortening, given the study populations in which they were carried out it is still unclear whether these findings are generalizable to the US population as a whole. Moreover, while prior research [32] has suggested that seropositivity for specific combinations of pathogens may play a more important role in the etiology of health outcomes than total number of pathogens, due to, for example, the potential for both antagonistic and synergistic pathogen interactions, understanding of which pathogens, alone or in combination, may cause the most damage to telomeres remains limited.

In the present study, we used data from a large, nationally representative sample of US adults to explore the assessment of pathogen burden captured by several different metrics. We assessed the cross-sectional associations between seropositivity for five persistent pathogens (HSV-1, HSV-2, CMV, *H.pylori*, and Hepatitis B)—analyzed as single pathogen associations, a pathogen burden summary score, and a latent class measure of pathogen burden accounting for both the total number and specific combination of pathogens for which individuals are seropositive, and leukocyte telomere length. Both the number and combination of pathogens may be more relevant to a given health outcome than either single pathogens or simply the total number of pathogens for which individuals are seropositive. Importantly, we also estimated these associations independent of key sociodemographic characteristics.

## Results

### Study sample

We used data from the National Health and Nutrition Examination Survey (NHANES), 1999–2000 survey. Our study sample included those with complete telomere, pathogen, and covariate data (*N* = 1708). Table 1 shows the population-weighted distributions of pathogen burden and telomere length for individuals aged 20–49 years by demographic and clinical characteristics. The greatest proportion of the study sample was in the 30–39 age group (38%) and 52% was female. The majority of the sample was Non-Hispanic White (70%) and 45% of the sample had a high school degree or less. Over half the sample (61%) was classified as overweight or obese (BMI greater than or equal to 25) and nearly half (47%) was a former or current smoker. The mean log telomere length for the total sample was 0.07 with differences by sociodemographic characteristic. We used the natural log of the T/S ratio to obtain a normally distributed measure of telomere length.

**Table 1:** Population-weighted distributions of pathogen burden and telomere length (log T/S ratio) for individuals aged 20–49 years by demographic and clinical characteristics, National Health and Nutrition Examination Survey (1999–2000), *n* = 1708

	Overall N (%)	Pathogen burden count score (%)				Pathogen burden composition class (%)			Leukocyte telomere length (log T/S/ ratio)
		0 (12%)	1 (27%)	2–3 (49%)	4+ (12%)	Comp 1 (49%)	Comp 2 (48%)	Comp 3 (3%)	Mean (SE)
**Overall**									0.07 (0.02)
**Age (years)**									
*20–29*	562 (30)	34	38	28	16	35	25	21	0.12 (0.02)
*30–39*	602 (38)	41	31	40	44	35	42	33	0.07 (0.02)
*40–49*	544 (32)	25	31	32	40	30	33	46	0.01 (0.03)
**Gender**									
*Male*	770 (48)	61	53	46	38	54	42	50	0.08 (0.02)
*Female*	938 (52)	39	47	54	62	46	58	50	0.06 (0.02)
**Race/Ethnicity**									
*N-H Black*	316 (10)	4	4	11	28	87	55	26	0.10 (0.02)
*N-H White*	724 (70)	89	88	65	32	4	17	11	0.02 (0.01)
*Hispanic*	627 (17)	4	7	21	35	7	25	56	0.06 (0.03)
*Other*	41 (3)	3	1	3	5	2	4	8	0.10 (0.06)
**Education**									
*< High school*	173 (4)	0.67	0.32	5	13	0	7	17	0.06 (0.03)
*Some high school*	340 (15)	6	9	18	27	9	21	26	0.06 (0.03)
*High school/GED*	398 (26)	24	26	28	22	26	26	36	0.05 (0.02)
*Some college/AA degree*	466 (30)	33	32	28	26	32	28	21	0.06 (0.02)
*College or above*	331 (25)	37	32	21	12	33	18	1	0.09 (0.03)
**Body Mass Index**									
*< 18.5*	28 (2)	3	1	3	4	2	3	0	0.04 (0.03)
*18.5 to < 25*	558 (36)	40	36	37	29	37	35	35	0.11 (0.02)
*25 to < 30*	562 (32)	23	37	33	31	33	33	27	0.06 (0.02)
*30+*	562 (29)	33	25	28	36	28	29	37	0.03 (0.03)
**Cigarette Smoking Status**									
*Never*	977 (53)	58	57	51	49	58	49	46	0.08 (0.02)
*Former*	288 (18)	16	19	18	18	17	18	18	0.04 (0.02)
*Current*	443 (29)	27	24	31	33	25	33	36	0.06 (0.02)

Comp 1 = Composition 1; Comp 2 = Composition 2; Comp 3 = Composition 3

N refers to unweighted sample sizes

We examined three separate pathogen measures: single pathogen associations, a pathogen count score and a latent class model of pathogen burden. Based on the latent class analysis, we classified individuals into one of three composition classes which reflect common pathogen co-occurrence, rather than only the sum of pathogens for which a person is positive. Nearly half of the sample (49%) was seropositive for 2–3 pathogens with large proportions seropositive to HSV-1 (63%) and CMV (56%) (Table 2). One quarter of the sample was seropositive to *H. pylori* (26%), 22% to HSV-2 and 15% to Hepatitis B. Forty-nine percent of the sample was assigned to composition class 1, a latent class characterized by moderate probabilities of HSV-1 and CMV and low probabilities of HSV-2, *H. pylori,* and Hepatitis B. Another 48% of the population was assigned to composition class 2, characterized by high probabilities of HSV-1 and CMV in addition to moderate probabilities of HSV-2 and *H.Pylori*. Finally, those assigned to composition class 3 (3% of the study population) had high probabilities of testing positive for HSV-1, CMV, and *H. pylori* with lower probabilities of HSV-2 and Hepatitis B.

**Table 2:** Population-weighted prevalence of individuals pathogens for individuals aged 20–49 years, National Health and Nutrition Examination Survey (1999–2000), *n* = 1708

Pathogen	N Positive	% Positive
HSV-1	1211	63.3
HSV-2	409	21.6
CMV	1149	56.2
*H.Pylori*	639	25.5
*Missing*	*N = 42*	
Hepatitis B	246	14.6

N refers to unweighted sample sizes. The percent values represent the proportion of the population that is positive for each of the listed pathogens

### Single pathogen associations

Using linear regression models, we examined associations between single pathogens and log telomere length. While generally infection with any single pathogen was associated with marginal increases in log telomere length, there were several cases where the opposite association was observed and no associations were statistically significant (see Supplemental Table 1).

For HSV-1, CMV, Hepatitis B, and *H. pylori*, infection was associated with lower log telomere length among those aged 30–39 years compared to either those aged 20–29 years or those aged 40–49 years. For HSV-2, infection was associated with lower log telomere length with increasing age. These associations were not statistically significant.

For HSV-2 and Hepatitis B, we found that infection was associated with lower log telomere length for males compared to females. For CMV and HSV-1, infection was associated with lower log telomere length for females. For *H. pylori*, there was no substantial difference in association between males and females. Again, these associations were not statistically significant.

### Pathogen burden summary score associations

We also examined associations between categories of the pathogen burden summary score and log telomere length. The distribution of the pathogen burden summary score by demographic and clinical characteristics is shown in Supplemental Table 2. We observed a general trend whereby relative to those in the 0 pathogen category, those in the 1 pathogen and 2–3 pathogens categories had a lower log telomere length in fully adjusted models for the overall sample, among those in the 30–39 year age group, among those in the 40–49 year age group, and among males. The associations were only statistically significant among males (Supplemental Table 3).

### Composition class associations

**Fig. 1:**
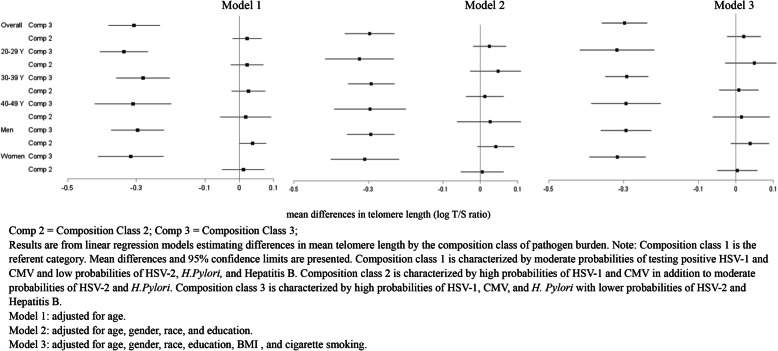
Population-weighted associations between total pathogen burden and mean telomere length (log T/S ratio) among individuals aged 20–49 years, National Health and Nutrition Examination Survey (1999–2000)

Lastly, we examined associations between each composition class and log telomere length. We observed consistent statistically significant associations between composition class 3 and log telomere length across all models (Fig. 1; Supplemental Table 6). Overall, those in composition class 3 (i.e., those with higher probabilities of testing positive for HSV-1, CMV, and *H. pylori* and lower probabilities of HSV-2 and Hepatitis B) had significantly lower log telomere length (− 0.30; 95% CI: − 0.36, − 0.24) compared to those in composition class 1 (moderate probabilities of HSV-1 and CMV and lower probabilities of the remaining three pathogens) in a model adjusting for age, gender, race/ethnicity, education, BMI, and cigarette smoking status.

The association between composition class 3 and log telomere length was also relatively consistent in sub-groups of age and gender. The association was largest among those in the 20–29 age group and females whereby those in composition class 3 had a lower log telomere length of 0.32 (95% CI: − 0.42, − 0.22 for 20–29 years; 95% CI: − 0.39, − 0.24 for females) compared to those in composition class 1 in a model controlling for age (in gender-stratified models), gender (in age-stratified models), race/ethnicity, education, BMI, and cigarette smoking status. We did not observe any statistically significant associations between composition class 2 and log telomere length, compared to composition class 1.

## Discussion

In this large, nationally representative study, we found associations between membership in composition class 3, a latent class characterized by high probabilities of infection with HSV-1, CMV, and *H. pylori*, and shorter telomere length. In contrast, we did not find consistent significant statistical associations with other measures of pathogen burden and telomere length. Importantly, the compositional classes differ from both single pathogen associations and pathogen burden summary score in that the composition classes account for both the number *and* combination of pathogens for which individuals are seropositive, whereas single pathogen associations only account for one pathogen and a summary score only accounts for the number of the pathogens. While the effect sizes with regards to the composition classes were modest and only observed in a small subset of the study population (i.e., findings only in composition class 3 which accounts for 3% of the study population), we feel that these findings are a novel contribution to the literature. Past research has largely focused on measuring pathogen burden as a simple count of the number of pathogens for which a person is seropositive without sufficiently accounting for contributions of certain combinations of pathogens. Thus, our study offers a key innovation in the measurement of pathogen burden. Further, the results from this study suggest that the biological effects of co-occurring persistent pathogens on accelerated cellular senescence patterns may begin early in the life course as we observed these patterns in a relatively young sample.

While our study found a statistically significant association between membership in composition class 3 and shorter telomere length, we did not find any associations with individual pathogens and shorter telomere length. The findings regarding infection with individual pathogens have been mixed. For example, in the study described above, Aiello et al. found a statistically significant association between CMV seropositivity and shortened telomeres but only among females [30]. Moreover, there were no observed associations between HSV-1, *H. pylori*, or *Chlamydia pneumonia* and telomere length. Another study by Dowd et al. using the Whitehall cohort, found that CMV infection (both seropositivity and higher CMV IgG antibody levels) was statistically significantly associated with declines in telomere length over time [31]. However, previous work in the Whitehall cohort reported no significant associations between CMV (seropositivity or CMV IgG antibody levels) infection and cross-sectional telomere length [33], consistent with our findings. In a study examining primary CMV infection, Van de Berg et al. found significant declines in telomere length following CMV infection in renal transplant patients [18].

It may be that both the younger age range of our study sample, the timing of pathogen measurement, and the cross-sectional nature of the current study contribute to differences in the observed findings. A hallmark of persistent viral infections is the reactivation of the infections across the life course. Therefore, associations between lifelong CMV reactivation may be more likely to be observed in an older study sample, such as the MESA sample where the mean age was between 55 and 58 years. Furthermore, unlike the Van de Berg study, we were unable to distinguish between primary and latent CMV infections. In our sample, it is likely that primary CMV infection happened earlier in the life course [34]. Additionally, only CMV seropositivity was measured in our NHANES study which did not match prior studies, which sometimes focused on the intensity of infection as measure by IgG antibody response. Finally, it may be that the relationship between CMV infection and telomere length cross-sectionally differs from what would be observed if telomere length was measured longitudinally.

An important strength of our analysis was the novel use of latent class analyses, which (to our knowledge) has not previously been used to examine the association between pathogen burden and telomere length. It may be that the composition classes that emerge through latent class analyses better capture population patterns of pathogen exposure history, which are more meaningful for predicting health outcomes than a count summary variable. Overall, identification of differences in telomere length by class of pathogen burden serves to shed light on which specific combinations of pathogens are most important in shaping telomere length. Meier et al. used a similar methodology as the current study—latent profile analyses of cumulative immune response to persistent infections—and found statistically significant associations between low educational attainment and membership in a high immune response category of pathogen burden [35]. Future studies utilizing methodologies for examining patterns of pathogen exposure may be able to further clarify the relationship between persistent infections and biological age patterns, such as telomere length.

There are several plausible biological mechanisms that could explain the relationship between persistent pathogen infection and telomere length. An aged immune phenotype that includes late-stage differentiated T cells and shortened telomeres are a commonly observed phenomenon in aging populations. It is plausible that chronic viral infections produce a similar phenotype observable in younger populations. Evidence from both human and animal studies suggest that the continual antigenic stimulation observed in chronic viral infections results in premature T cell exhaustion with corresponding accelerated telomere shortening [36]. Studies directly examining the circulating T cell pool have found that after primary CMV infection there is an increase in late-stage differentiated T cells (both CD4 and CD8), consistent with significant decreases in lymphocyte telomere length [18]. This same pattern likely holds true for many persistent infections, such as herpesvirus infections. In a prospective study of telomere length in the British Whitehall cohort, Dowd et al. reported statistically significant declines in telomere length over a three year period associated with infection with several herpsesvirus coinfections, with CMV as the driving force behind these associations [31]. The implication of such studies is that persistent infections are a causal mechanism for accelerated biological age patterns and that CMV plays a role in the majority of studies to date.

There are a few limitations to this study that should also be considered. While we believe the young age of the sample is a strength of this study in that far fewer studies have focused on younger as opposed to older individuals, it may also be a limitation given the low variation in telomere length among younger age groups. Additionally, questions remain as to the temporal direction of the relationship between telomere length and persistent infections given the cross-sectional nature of our sample. Indeed, several prospective studies also suggest the possibility of reverse causality in that shortened telomere length may increase risk of infection, and clinical disease following infection. For example, in experimental studies of Rhinovirus infection, Cohen et al. found that shortened telomere length in lymphocytes (specifically, CD4 and CD8 cells) was associated with greater risk of infection given uniform exposure to a pathogen [37]. Notably, their studies were done among a young, healthy population. A 2017 study documented that blood leukocyte telomere length at baseline was associated with higher risk of any infection, and specifically pneumonia during 23 years of follow-up [38]. Given our study was cross-sectional, additional longitudinal studies are needed to further clarify the direction of the relationship between infections and cellular aging. Finally, classifying individuals as seropositive/seronegative to a specific infection may result in a loss of key information regarding the overall morbidity from a given infection or combination of infection. While only data on seropositivity was available in NHANES, future studies would greatly benefit from the use of continuous antibody levels to infection, since immune response to chronic infection has been shown to be a more salient determinant of many age-related conditions [39].

Finally, the observed results may have been biased by unmeasured confounders. While we employed a broad set of control variables in the regression models that we believe are salient indicators of the social environment, there may be other unmeasured variables that bias the observed association.

## Conclusions

Despite these limitations, we believe the findings of this study are a critical contribution to the body of literature examining the link between persistent infections and telomere length for several reasons. First, we were able to discern differences in telomere length associated with patterns of pathogen exposure in a relatively young, US-representative sample. Studying these associations before the onset of many age-related diseases is a critical step in addressing the evolution of pathogens, immunity and aging. Second, we were able to operationalize pathogen burden in multiple ways, allowing us to better discern the ways in which pathogen burden may be impacting health across the life course.

Telomere length has been widely cited as a measure of cellular aging that is impacted by social, environmental, and biological processes. Furthermore, shortened telomere length has been linked to a number of outcomes in late life including atherosclerosis and cardiovascular disease [4, 5]. Persistent infections and the subsequent life-long immune response to these infections may be key drivers of cellular age patterns such as telomere length. Future research should consider assessing not only seropositivity but also indicators of immune response to individual as well as specific combinations of persistent pathogens in relation to telomere length and ultimately the onset of age-related conditions.

## Methods

The National Health and Nutrition Examination Survey (NHANES) is a nationally representative, cross-sectional survey of the non-institutionalized US population conducted by the Centers for Disease Control and Prevention (CDC). Our study sample consisted of those individuals who participated in the 1999–2000 wave and who contributed survey, physical examination, and laboratory data. Further details on the study design are provided in the references [40].

**Fig. 2:**
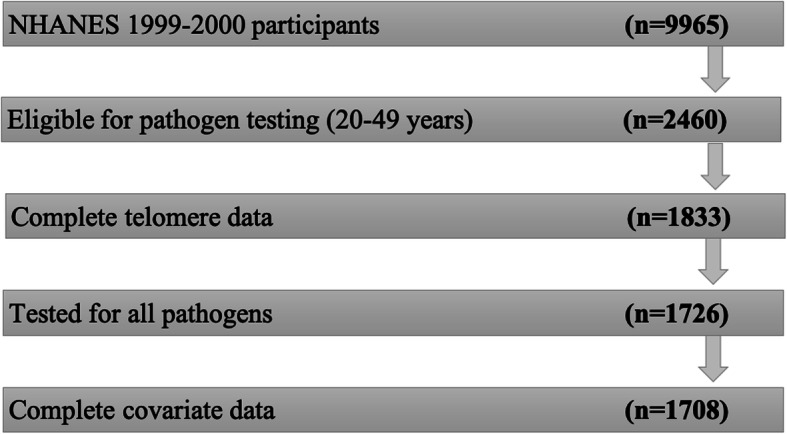
Description of the study sample composition for the National Health and Nutrition Examination Survey, 1999–2000, *n* = 1708

Of the 9965 respondents in the NHANES 1999–2000 sample, our analysis included the 1708 individuals aged 20–49 years with complete telomere, pathogen, and covariate data (Fig. 2). Importantly, we excluded individuals greater than 49 years of age because those individuals did not undergo pathogen testing. All analyses accounted for the NHANES complex survey design and non-response to obtain nationally representative estimates. Analyses were done in STATA 14 (StataCorp, LLC, College Station, TX) using the ‘*svy’* package with the subpop option and SAS v9.4 (SAS Institute, Inc., Cary, NC).

### Leukocyte telomere length

Leukocyte telomere length was measured in participant blood sera using the quantitative polymerase chain reaction (qPCR) method to measure length relative to a standard reference DNA. This provides a continuous T/S ratio value as described previously [15, 41]. The NHANES provides a mean T/S ratio corresponding to the mean of three T/S ratio values calculated for each sample.

For analytic purposes, we utilized the log of the mean T/S ratio in order to approximate a normal distribution and treated this variable as continuous. Additional details on the measurement of leukocyte telomere length are included in the supplementary material.

### Pathogens

We used data on five persistent pathogens that were tested in the 1999–2000 wave: HSV-1, HSV-2, CMV, *H. pylori*, and Hepatitis B. Immunoglobulin G (IgG) responses to each pathogen were measured in participant blood sera and categorized as seropositive or seronegative based on the cut-points designated by NHANES. Additional details on the measurement of each pathogen are included in the supplementary material. Details on the laboratory testing procedures for each pathogen can be found in the CDC NHANES Laboratory Data documentation [42]. Of important note, widespread vaccination efforts against Hepatitis B began in the early 1990s in the US with a focus on universal vaccination among children. Our study population would not have been included in the widespread vaccination campaigns and thus we expect little confounding by vaccination status in our analyses of Hepatitis B.

### Pathogen burden measurement

We constructed two different measures of pathogen burden: one based on the summed number of pathogens for which individuals were seropositive and one based on a latent class model.

The pathogen burden summary score was based on the summed total number of pathogens of pathogens for which an individual was seropositive (range 0–5) and was categorized as 0 pathogens, 1 pathogen, 2–3 pathogens, and 4+ pathogens. Those with 0 pathogens served as the referent category.

We also used a latent class analysis (LCA) to measure pathogen burden. LCA creates groups (i.e., classes) based on common pathogen co-occurrence, as well as allows the effect of LCA group membership on the outcome to vary. For this reason, LCA may be able to identify patterns of pathogen infection in the study population which may be more relevant to a given health outcome than either single pathogens or simply the total number of pathogens for which individuals are seropositive. Detailed information on the latent class approach for classifying pathogen burden is provided in the supplementary material. Briefly, we used the three-step inclusive classify-analyze approach [43] to assign individuals with similar profiles of pathogen seropositivity to mutually exclusive classes on both manifest (measured) and latent (unmeasured) characteristics. Latent class models were estimated using PROC LCA (Lanza et al. 2015 in SAS Version 9.4). Model fit statistics were assessed for latent class models with two to five latent classes. A five-class solution did not converge. Based on both model fit indices and interpretability, a three-class solution was deemed appropriate as it optimized Akaike information criteria (AIC), log-likelihood, and entropy values (see supplemental Table 5). We then assigned individuals to a specific latent class based on their posterior probability of most likely class membership. We compared the distribution of pathogens in the pathogen burden summary score and latent class analyses. Results are shown in supplemental Table 6 and supplemental Figure 1.

**Fig. 3:**
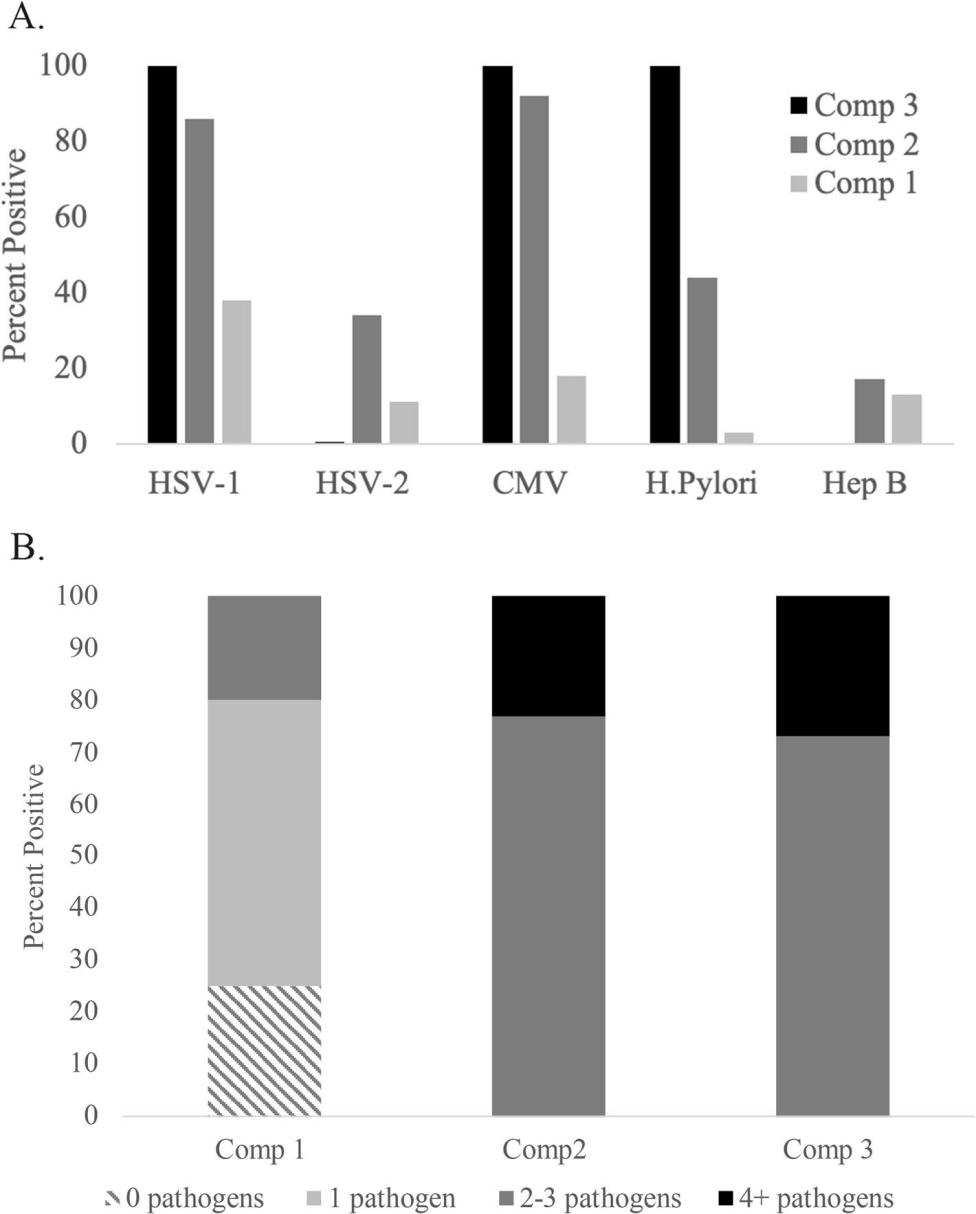
**a** Conditional probability of testing positive for a specific pathogen given composition class membership, National Health and Nutrition Examination Survey (1999–2000), n = 1708. **b**. Population-weighted distributions of pathogen compositional class by pathogen count score, National Health and Nutrition Examination Survey (1999–2000), n = 1708

Conditional-response probabilities from the three-class latent model are presented in Fig. 3a. Composition class 1 (49% of the sample) was characterized by moderate probabilities of HSV-1 and CMV but lower probabilities of the remaining three pathogens. We consider this class to be characterized by infection with common persistent pathogens. Composition class 2 was characterized by higher probabilities of HSV-1 and CMV and moderate probabilities of HSV-2 and *H.Pylori*. Finally, composition class 3 (3% of the sample) had higher probabilities of testing positive for HSV-1, CMV, and *H. pylori* with lower probabilities of HSV-2 and Hepatitis B. Individuals in this class appear to experience a high burden of persistent pathogens. Figure 3b shows the distribution of the composition classes by pathogen count category. The variation in log telomere length for each individual pathogen as well as the pathogen burden composition classes is shown in Supplemental Figure 2.

### Covariates

We also included several covariates that we believe are salient indicators of key sociodemographic and health behavior characteristics. Covariates included age, gender, race/ethnicity, education, body mass index (BMI) (kg/m^2^), and cigarette smoking status. Age was modelled continuously in years.

Gender was categorized as male or female. Race/ethnicity included the following four categories: Non-Hispanic White, Non-Hispanic Black, Hispanic, and Other. Hispanic included both those classified as Mexican American as well as Other Hispanic. Educational attainment was measured by five categories: below high school, some high school, high school/GED, some college, and college or above. We divided BMI (kg/m^2^) into four categories based on guidelines from the CDC: < 18.5, 18.5- < 25, 25.0- < 30, and 30.0 or higher. Cigarette smoking status was classified into three categories: never smoker, former smoker, and current smoker. Covariates were categorized as recommended in the NHANES analytic guidelines unless otherwise noted.

### Regression analyses

We assessed associations of single pathogens, pathogen burden summary score, and the pathogen burden latent classes (hereafter referred to as composition classes) with log telomere length using covariate-adjusted linear regression. Importantly, we also examined associations controlling for several key covariates in order to estimate the associations independent of sociodemographic characteristics. Model 1 was adjusted for age only. Model 2 was adjusted for age, gender, race, and education level. Model 3 was adjusted for age, gender, race, education, BMI, and cigarette smoking status. Based on known differences in telomere length by gender and age [44, 45], we also stratified analyses by both gender and age group (i.e., aged 20–29, 30–39, and 40–49 years). These analyses included the adjustments sets outlined above. For all analyses, a two-side *p*-value of 0.05 was used to determine statistical significance.

## Supplementary Information

**Additional file 1.**

## Data Availability

The dataset supporting the conclusions of this article is derived from the National Health and Nutrition Examination Survey and are publicly available through the U.S. Centers for Disease Control and Prevention.

## Abbreviations

BMI: Body mass index
CDC: Centers for Disease Control and Prevention
CMV: Cytomegalovirus
*H. pylori*: *Helicobacter pylori*
HSV: Herpes simplex virus
IgG: Immunoglobulin G
LCA: Latent class analysis
MESA: Multiethnic Study of Atherosclerosis
NHANES: National Health and Nutrition Examination Survey
qPCR: Quantitative polymerase chain reaction
