# Supplementary material for: Pathogen burden and leukocyte telomere length in the United States
Source: Immun Ageing. 2020 Nov 19;17:36. doi: 10.1186/s12979-020-00206-9 (PMC7677839; doi:10.1186/s12979-020-00206-9)
Supplement: Supplementary file 1 — Additional file 1. [file 12979_2020_206_MOESM1_ESM.docx]

**Pathogen Burden and Leukocyte Telomere Length in the United States**

***Supplemental Material***

**Measurement of Pathogens**

We used data on five persistent pathogens that were tested in the 1999-2000 wave: herpes simplex virus type 1 (HSV-1), herpes simplex virus type 2 (HSV-2), Cytomegalovirus (CMV) *Helicobacter pylori (H.* *pylori)*, and Hepatitis B. Immunoglobulin G (IgG) responses to each pathogen were measured in participant blood sera and categorized as seropositive or seronegative based on the cut-points designated by NHANES ^1-3^. CMV positivity was determined based on blood serum CMV specific IgG antibodies measured with an ELISA. Optical density units were reported and classified as positive, negative, or equivocal. Seropositivity for HSV-1 and HSV-2 was determined based on immunodot assay using the glycoprotein gG-1 antigen. *H. pylori* was determined based on the presence of IgG antibodies in human serum detected by an IgG ELISA. Chlamydia was characterized by the presence of *Chlamydia trachomatis* in the urine. Infection was determined using the ligase chain reaction (LCR) amplification to detect the presence of *Chlamydia trachomatis* DNA. Hepatitis B infection was determined based the presence of anti-HBs levels in serum or plasma using a solid phase ELISA.

**Leukocyte Telomere Length:**

Leukocyte telomere length was determined based on an assay using the quantitative PCR method that measures telomere length relative to a standard reference DNA, generating the T/S ratio. The assay was performed in the Blackburn lab at the University of California, San Francisco. Each blood sample was assayed 3 times on 3 different days. The mean and standard deviation T/S ratio for the samples were calculated.

**Latent Class Analyses:**

We used a three-step inclusive classify-analyze approach for the latent class analysis ^4^. This method assigns individuals with similar profiles of pathogen seropositivity to mutually exclusive classes on both measured and latent (unmeasured) characteristics. This method assumes there an underlying total pathogen burden an individual experiences and thus seropositivity to any one pathogen is a signal of the underlying burden. The three-step inclusive classify-analyze approach separates the classification of individuals into latent classes from the regression of the outcome on the latent classes.
 Following this approach, we first determined the appropriate number of latent classes for each wave by comparing model fit indices: the likelihood-ratio value (G2), Akaike information criteria (AIC), Bayesian information criterion (BIC), sample size-adjusted BIC, log-likelihood, and entropy. Lower values for the AIC, BIC, adjusted AIC are preferred as they indicate better model fit. Higher values of entropy indicate greater distinction between the classes. When deciding on the optimal number of classes, we prioritized both model fit and interpretability of the classes. Model estimation was repeated 1,000 times with random starts to confirm model identification. Latent class models were estimated using PROC LCA (Lanza et al. 2015 in SAS Version 9.4).
 Once the appropriate number of latent classes was determined, we assigned individuals to a specific latent class based on their posterior probability of most likely class membership. Following the inclusive classify-analyze approach, the outcome variable, the telomere length, was used as a covariate in the final model to reduce bias in estimation in the outcome model.^4^ We then regressed telomere length on the latent classes to determine if latent class membership was related to telomere length when controlling for other covariates.

**References:**

1. Lennette EH, Balows A. *Manual of clinical microbiology 4 Ed.* 1985.

2. Ashley RL, Militoni J, Lee F, Nahmias A, Corey L. Comparison of Western blot (immunoblot) and glycoprotein G-specific immunodot enzyme assay for detecting antibodies to herpes simplex virus types 1 and 2 in human sera. *Journal of clinical microbiology.* 1988;26(4):662-667.

3. Lee FK, Pereira L, Griffin C, Reid E, Nahmias A. A novel glycoprotein for detection of herpes simplex virus type 1-specific antibodies. *Journal of virological methods.* 1986;14(2):111-118.

4. Bray BC, Lanza ST, Tan X. Eliminating Bias in Classify-Analyze Approaches for Latent Class Analysis. [*https://doiorg/101080/107055112014935265*](https://doiorg/101080/107055112014935265)*.* 2014.

**Supplemental Table 1.** Population-weighted associations between individual pathogen seropositivity and log telomere length among individuals aged 20-49 years, National Health and Nutrition Examination Survey (1999-2000).

|  |  |  | | | **β (95% Confidence Interval) for decrease in log-telomere length** | | |  |
| --- | --- | --- | --- | --- | --- | --- | --- | --- |
|  | **Model 1** | | **P** | **Model 2** | | **P** | **Model 3** | **P** |
| **Overall** |  | |  |  | |  |  |  |
| hsv1 positive versus negative | -0.01 (-0.03, 0.02) | | 0.71 | 0 (-0.03, 0.03) | | 0.92 | 0.002 (-0.03, 0.03) | 0.90 |
| hsv2 positive versus negative | 0.00 (-0.04, 0.04) | | 0.997 | 0.00 (-0.04, 0.04) | | 0.99 | -0.0001 (-0.04, 0.04) | 0.99 |
| cmv positive versus negative | 0.00 (-0.03, 0.03) | | 0.95 | 0.01 (-0.02, 0.04) | | 0.56 | 0.006 (-0.03, 0.04) | 0.68 |
| hp positive versus negative | 0.01 (-0.03, 0.05) | | 0.60 | 0.02 (-0.03, 0.06) | | 0.42 | 0.01 (-0.03, 0.05) | 0.51 |
| hepb positive versus negative | -0.01 (-0.04, 0.02) | | 0.50 | -0.01 (-0.04, 0.02) | | 0.39 | -0.02 (-0.05, 0.01) | 0.28 |
| **Age 20-29** |  | |  |  | |  |  |  |
| hsv1 positive versus negative | -0.01 (-0.04, 0.03) | | 0.60 | 0.01 (-0.03, 0.04) | | 0.71 | 0.004 (-0.03, 0.04) | 0.81 |
| hsv2 positive versus negative | 0.01 (-0.05, 0.07) | | 0.71 | 0.03 (-0.02, 0.08) | | 0.18 | 0.03 (-0.01, 0.08) | 0.13 |
| cmv positive versus negative | 0.01 (-0.03, 0.04) | | 0.73 | 0.03 (-0.02, 0.08) | | 0.29 | 0.03 (-0.02, 0.08) | 0.27 |
| hp positive versus negative | 0.00 (-0.05, 0.05) | | 0.93 | 0.01 (-0.07, 0.08) | | 0.81 | 0.01 (-0.07, 0.09) | 0.73 |
| hepb positive versus negative | -0.01 (-0.07, 0.04) | | 0.60 | -0.02 (-0.08, 0.05) | | 0.61 | -0.01 (-0.07, 0.05) | 0.66 |
| **Age 30-39** |  | |  |  | |  |  |  |
| hsv1 positive versus negative | -0.01 (-0.07, 0.06) | | 0.86 | -0.01 (-0.07, 0.05) | | 0.70 | -0.006 (-0.07, 0.06) | 0.84 |
| hsv2 positive versus negative | 0.01 (-0.04, 0.05) | | 0.79 | 0 (-0.05, 0.05) | | 0.86 | -0.001 (-0.04, 0.04) | 0.96 |
| cmv positive versus negative | 0.01 (-0.04, 0.05) | | 0.75 | 0 (-0.04, 0.03) | | 0.93 | -0.005 (-0.05, 0.04) | 0.82 |
| hp positive versus negative | 0.02 (-0.03, 0.07) | | 0.40 | 0.01 (-0.06, 0.07) | | 0.68 | 0.005 (-0.06, 0.07) | 0.85 |
| hepb positive versus negative | -0.03 (-0.08, 0.01) | | 0.17 | -0.04 (-0.09, 0.00) | | 0.11 | -0.04 (-0.09, 0.001) | 0.06 |
| **Age 40-49** |  | |  |  | |  |  |  |
| hsv1 positive versus negative | 0 (-0.07, 0.06) | | 0.89 | 0.01 (-0.06, 0.08) | | 0.75 | 0.007 (-0.06, 0.07) | 0.82 |
| hsv2 positive versus negative | -0.01 (-0.07, 0.05) | | 0.71 | 0 (-0.07, 0.06) | | 0.85 | -.02 (-0.07, 0.04) | 0.54 |
| cmv positive versus negative | -0.01 (-0.07, 0.05) | | 0.75 | 0.01 (-0.06, 0.08) | | 0.78 | 0.003 (-0.06, 0.06) | 0.92 |
| hp positive versus negative | 0.01 (-0.06, 0.07) | | 0.88 | 0.03 (-0.04, 0.1) | | 0.61 | 0.009 (-0.06, 0.08) | 0.76 |
| hepb positive versus negative | 0.02 (-0.06, 0.09) | | 0.60 | 0.02 (-0.04, 0.08) | | 0.60 | 0.01 (-0.06, 0.08) | 0.73 |
| **Females** |  | |  |  | |  |  |  |
| hsv1 positive versus negative | -0.01 (-0.05, 0.04) | | 0.72 | 0.00 (-0.05, 0.04) | | 0.85 | -0.004 (-0.05, 0.04) | 0.84 |
| hsv2 positive versus negative | 0.01 (-0.05, 0.07) | | 0.76 | 0.00 (-0.06, 0.07) | | 0.95 | 0.004 (-0.06, 0.06) | 0.89 |
| cmv positive versus negative | -0.01 (-0.07, 0.04) | | 0.65 | -0.02 (-0.06, 0.03) | | 0.50 | -0.02 (-0.06, 0.03) | 0.45 |
| hp positive versus negative | 0.02 (-0.02, 0.06) | | 0.40 | 0.02 (-0.02, 0.06) | | 0.28 | 0.01 (-0.02, 0.05) | 0.45 |
| hepb positive versus negative | 0.00 (-0.05, 0.05) | | 0.9995 | 0.00 (-0.05, 0.05) | | 0.97 | -0.005 (-0.05, 0.04) | 0.83 |
| **Males** |  | |  |  | |  |  |  |
| hsv1 positive versus negative | 0.00 (-0.03, 0.03) | | 0.94 | 0.00 (-0.03, 0.04) | | 0.86 | 0.004 (-0.03, 0.04) | 0.79 |
| hsv2 positive versus negative | -0.01 (-0.06, 0.04) | | 0.75 | 0.00 (-0.06, 0.05) | | 0.83 | -0.007 (-0.06, 0.04) | 0.76 |
| cmv positive versus negative | 0.02 (-0.03, 0.06) | | 0.43 | 0.03 (-0.02, 0.07) | | 0.23 | 0.03 (-0.02, 0.07) | 0.28 |
| hp positive versus negative | 0.00 (-0.06, 0.06) | | 0.89 | 0.01 (-0.06, 0.09) | | 0.70 | 0.009 (-0.06, 0.08) | 0.78 |
| hepb positive versus negative | -0.02 (-0.06, 0.02) | | 0.32 | -0.02 (-0.07, 0.02) | | 0.23 | -0.03 (-0.07, 0.01) | 0.17 |

*P value <0.05

Model 1: Adjusted for age

Model 2: Adjusted for age, gender, race, education

Model 3: Adjusted for age, gender, race, education, BMI, cigarette smoking status, CRP, white blood cell count

**Supplemental Table 2.** Distribution of the pathogen burden summary score for individuals aged 20-49 years by demographic and clinical characteristics and telomere length (log T/S ratio), n = 1,708.

|  | **Pathogen Count**  **N (%)** | | | | | | | |
| --- | --- | --- | --- | --- | --- | --- | --- | --- |
|  | 0  147 (12) | 1  339 (27) | 2  423 (27) | 3  485 (22) | 4  236 (9) | 5  68 (2) | 6  10 (0.5) | |
| **Age (years)** |  |  |  |  |  |  |  | |
| *20-29* | 34 | 38 | 29 | 26 | 19 | 2 | 23 | |
| *30-39* | 41 | 31 | 39 | 41 | 46 | 42 | 22 | |
| *40-49* | 25 | 31 | 32 | 33 | 35 | 56 | 55 | |
| **Gender** |  |  |  |  |  |  |  | |
| *Male* | 61 | 53 | 50 | 40 | 35 | 37 | 88 | |
| *Female* | 39 | 47 | 50 | 60 | 65 | 63 | 12 | |
| **Race/Ethnicity** |  |  |  |  |  |  |  | |
| *N-H Black* | 4 | 4 | 8 | 14 | 22 | 49 | 33 | |
| *N-H White* | 89 | 88 | 75 | 52 | 35 | 28 | 0 | |
| *Hispanic* | 4 | 8 | 14 | 30 | 38 | 17 | 67 | |
| *Other* | 3 | 1 | 1 | 4 | 5 | 6 | 0 | |
| **Education** |  |  |  |  |  |  |  | |
| *<High school* | 0.70 | 0.32 | 3 | 8 | 13 | 12 | 18 | |
| *Some high school* | 6 | 9 | 14 | 23 | 26 | 28 | 46 | |
| *High school/GED* | 24 | 26 | 29 | 26 | 21 | 24 | 28 | |
| *Some college/AA degree* | 33 | 33 | 29 | 27 | 28 | 22 | 9 | |
| *College or above* | 37 | 34 | 25 | 16 | 13 | 14 | 0 | |
| **Body Mass Index** |  |  |  |  |  |  |  | |
| *<18.5* | 3 | 1 | 2 | 4 | 5 | 0 | 0 | |
| *18.5 to <25* | 40 | 36 | 34 | 40 | 30 | 25 | 28 | |
| *25 to <30* | 23 | 37 | 35 | 30 | 28 | 34 | 68 | |
| *30+* | 33 | 25 | 30 | 26 | 37 | 41 | 4 | |
| **Cigarette Smoking Status** | | | | | | | |  |
| *Never* | 58 | 57 | 53 | 50 | 47 | 54 | 67 | |
| *Former* | 16 | 19 | 19 | 15 | 22 | 5 | 5 | |
| *Current* | 27 | 24 | 28 | 35 | 32 | 41 | 28 | |
| **Log Telomere Length**  *Mean (SE)* | 0.10 (0.03) | 0.07 (0.02) | 0.06 (0.02) | 0.05 (0.03) | 0.07 (0.03) | 0.10 (0.06) | 0.14 (0.05) | |
| N refers to unweighted sample sizes. Weighted frequencies are reported for categorical variables. Weighted means and standard errors are reported for continuous variables. | | | | | | | | |

**Supplemental Table 3.** Population-weighted associations between pathogen burden summary score and log telomere length among individuals aged 20-49 years, NHANES (1999-2000).

|  |  | **β (95% Confidence Interval) for decrease in log-telomere length** | | | | | |
| --- | --- | --- | --- | --- | --- | --- | --- |
|  | **N** | **Model 1** | **P** | **Model 2** | **P** | **Model 3** | **P** |
| **Overall** |  |  |  |  |  |  |  |
| Count category |  |  |  |  |  |  |  |
| *0 pathogens* | 147 | REF |  | REF |  | REF |  |
| *1 pathogen* | 339 | -0.03 (-0.08, 0.02) | 0.19 | -0.03 (-0.07, 0.02) | 0.24 | -0.03 (-0.07, 0.02) | 0.21 |
| *2-3 pathogens* | 908 | -0.04 (-0.08, 0.01) | 0.12 | -0.02 (-0.07, 0.02) | 0.30 | -0.03 (-0.07, 0.02) | 0.24 |
| *4+ pathogens* | 314 | 0 (-0.07, 0.06) | 0.95 | 0.01 (-0.04, 0.06) | 0.72 | 0 (-0.04, 0.05) | 0.82 |
| **Age 20-29** |  |  |  |  |  |  |  |
| Count category |  |  |  |  |  |  |  |
| *0 pathogens* | 63 | REF |  | REF |  | REF |  |
| *1 pathogen* | 142 | -0.01 (-0.06, 0.04) | 0.70 | 0 (-0.06, 0.06) | 0.96 | 0 (-0.06, 0.06) | 0.91 |
| *2-3 pathogens* | 304 | -0.01 (-0.07, 0.04) | 0.61 | 0.01 (-0.07, 0.10) | 0.73 | 0.02 (-0.07, 0.11) | 0.68 |
| *4+ pathogens* | 53 | -0.01 (-0.12, 0.10) | 0.85 | 0.01 (-0.11, 0.13) | 0.83 | 0.02 (-0.10, 0.14) | 0.75 |
| **Age 30-39** |  |  |  |  |  |  |  |
| Count category |  |  |  |  |  |  |  |
| *0 pathogens* | 56 | REF |  | REF |  | REF |  |
| *1 pathogen* | 102 | -0.04 (-0.13, 0.04) | 0.28 | -0.05 (-0.13, 0.03) | 0.22 | -0.05 (-0.13, 0.04) | 0.24 |
| *2-3 pathogens* | 327 | -0.03 (-0.11, 0.05) | 0.43 | -0.04 (-0.11, 0.04) | 0.30 | -0.04 (-0.12, 0.04) | 0.34 |
| *4+ pathogens* | 117 | -0.02 (-0.12, 0.09) | 0.71 | -0.04 (-0.14, 0.05) | 0.35 | -0.04 (-0.14, 0.05) | 0.34 |
| **Age 40-49** |  |  |  |  |  |  |  |
| Count category |  |  |  |  |  |  |  |
| *0 pathogens* | 28 | REF |  | REF |  | REF |  |
| *1 pathogen* | 95 | -0.04 (-0.13, 0.05) | 0.32 | -0.04 (-0.11, 0.04) | 0.32 | -0.04 (-0.10, 0.03) | 0.25 |
| *2-3 pathogens* | 277 | -0.07 (-0.16, 0.02) | 0.13 | -0.04 (-0.10, 0.03) | 0.25 | -0.04 (-0.10, 0.01) | 0.10 |
| *4+ pathogens* | 144 | 0 (-0.09, 0.10) | 0.92 | 0.05 (-0.04, 0.14) | 0.25 | 0.04 (-0.04, 0.12) | 0.29 |
| **Females** |  |  |  |  |  |  |  |
| Count category |  |  |  |  |  |  |  |
| *0 pathogens* | 67 | REF |  | REF |  | REF |  |
| *1 pathogen* | 167 | 0.04 (-0.05, 0.12) | 0.41 | 0.04 (-0.05, 0.13) | 0.38 | 0.04 (-0.06, 0.13) | 0.42 |
| *2-3 pathogens* | 511 | 0.02 (-0.09, 0.13) | 0.76 | 0.02 (-0.09, 0.13) | 0.71 | 0.02 (-0.10, 0.13) | 0.77 |
| *4+ pathogens* | 193 | 0.03 (-0.09, 0.15) | 0.61 | 0.03 (-0.08, 0.13) | 0.62 | 0.02 (-0.08, 0.12) | 0.63 |
| **Males** |  |  |  |  |  |  |  |
| Count category |  |  |  |  |  |  |  |
| *0 pathogens* | 80 | REF |  | REF |  | REF |  |
| *1 pathogen* | 172 | **-0.08 (-0.13, -0.03)** | **0.005** | **-0.08 (-0.12, -0.03)** | **0.002** | **-0.08 (-0.12, -0.04)** | **0.001** |
| *2-3 pathogens* | 397 | **-0.06 (-0.10, -0.03)** | **0.002** | **-0.05 (-0.10, -0.01)** | **0.01** | **-0.06 (-0.10, -0.02)** | **0.006** |
| *4+ pathogens* | 121 | 0 (-0.06, 0.06) | 0.92 | 0.01 (-0.05, 0.07) | 0.67 | 0.01 (-0.05, 0.06) | 0.73 |
| N refers to unweighted sample sizes. Bolded values reflect statistical significance at P < 0.05.  Model 1: Adjusted for age  Model 2: Adjusted for age, gender, race, education  Model 3: Adjusted for age, gender, race, education, BMI, and cigarette smoking status. | | | | | | | |

**Supplemental Table 4.** Population-weighted associations between compositional classes of pathogen burden and log telomere length among individuals aged 20-49 years, NHANES (1999-2000).

|  |  | **β (95% Confidence Interval) for decrease in log-telomere length** | | | | | |
| --- | --- | --- | --- | --- | --- | --- | --- |
|  | **N** | **Model 1** | **P** | **Model 2** | **P** | **Model 3** | **P** |
| **Overall** |  |  |  |  |  |  |  |
|  |  |  |  |  |  |  |  |
| *Comp 1* | 614 | REF |  | REF |  | REF |  |
| *Comp 2* | 990 | 0.02 (-0.02, 0.06) | 0.26 | 0.02 (-0.02, 0.07) | 0.26 | 0.02 (-0.02, 0.07) | 00.33 |
| *Comp 3* | 104 | **-0.31 (-0.38, -0.23)** | **<0.0001** | **-0.30 (-0.36, -0.23)** | **<0.0001** | **-0.30 (-0.36, -0.24)** | **<0.0001** |
|  |  |  |  |  |  |  |  |
| **Age 20-29** |  |  |  |  |  |  |  |
| *Comp 1* | 246 | REF |  | REF |  | REF |  |
| *Comp 2* | 291 | 0.02 (-0.02, 0.07) | 0.32 | 0.05 (-0.03, 0.12) | 0.19 | 0.05 (-0.03, 0.13) | 0.19 |
| *Comp 3* | 25 | **-0.34 (-0.41, -0.27)** | **<0.0001** | **-0.32 (-0.42, -0.23)** | **<0.0001** | **-0.32 (-0.42, -0.22)** | **<0.0001** |
|  |  |  |  |  |  |  |  |
| **Age 30-39** |  |  |  |  |  |  |  |
| *Comp 1* | 205 | REF |  | REF |  | REF |  |
| *Comp 2* | 369 | 0.03 (-0.02, 0.08) | 0.26 | 0.01 (-0.04, 0.06) | 0.62 | 0.01 (-0.05, 0.06) | 0.75 |
| *Comp 3* | 28 | **-0.28 (-0.36, -0.20)** | **<0.0001** | **-0.29 (-0.35, -0.23)** | **<0.0001** | **-0.29 (-0.35, -0.23)** | **<0.0001** |
|  |  |  |  |  |  |  |  |
| **Age 40-49** |  |  |  |  |  |  |  |
| *Comp 1* | 163 | REF |  | REF |  | REF |  |
| *Comp 2* | 330 | 0.02 (-0.06, 0.09) | 0.59 | 0.03 (-0.06, 0.12) | 0.54 | 0.01 (-0.06, 0.09) | 0.68 |
| *Comp 3* | 51 | **-0.31 (-0.42, -0.20)** | **<0.0001** | **-0.30 (-0.39, -0.20)** | **<0.0001** | **-0.29 (-0.39, -0.20)** | **<0.0001** |
|  |  |  |  |  |  |  |  |
| **Females** |  |  |  |  |  |  |  |
| *Comp 1* | 315 | REF |  | REF |  | REF |  |
| *Comp 2* | 403 | 0.01 (-0.05, 0.07) | 0.69 | 0.005 (-0.05, 0.06) | 0.87 | 0.004 (-0.05, 0.06) | 0.89 |
| *Comp 3* | 52 | **-0.32 (-0.41, -0.22)** | **<0.0001** | **-0.31 (-0.40, -0.22)** | **<0.0001** | **-0.32 (-0.39, -0.24)** | **<0.0001** |
|  |  |  |  |  |  |  |  |
| **Males** |  |  |  |  |  |  |  |
| *Comp 1* | 299 | REF |  | REF |  | REF |  |
| *Comp 2* | 587 | 0.04 (0.001, 0.08) | 0.05 | 0.04 (-0.01, 0.09) | 0.10 | 0.04 (-0.01, 0.09) | 0.13 |
| *Comp 3* | 52 | **-0.30 (-0.37, -0.22)** | **<0.0001** | **-0.29 (-0.36, -0.23)** | **<0.0001** | **-0.29 (-0.36, -0.23)** | **<0.0001** |
| N refers to unweighted sample sizes.  Bolded values reflect statistical significance at P < 0.05.  Model 1: Adjusted for age  Model 2: Adjusted for age, gender, race, education  Model 3: Adjusted for age, gender, race, education, BMI, and cigarette smoking status. | | | | | | | |

**Supplemental Table 5.** Model fit statistics for latent class models of pathogen burden compositional classes (N = 1, 708).

| **Number of Classes** | | **G^2^** | ***df*** | **AIC** | **BIC** | **Adjusted BIC** | **Log-Likelihood** | **Entropy** |
| --- | --- | --- | --- | --- | --- | --- | --- | --- |
|  |  | |  |  |  |  |  |  |
| 2 | 49.58 | | 20 | 71.58 | 131.48 | 96.53 | -4666.23 | 0.54 |
| **3** | **24.64** | | **14** | **58.64** | **151.2** | **97.19** | **-4653.76** | **0.63** |
| 4 | 15.87 | | 8 | 61.87 | 187.1 | 114.03 | **-**4649.38 | 0.55 |
| Note: a 5 class model did not converge | | | |  |  |  |  |  |

**Supplemental Table 6.** Distribution of the study population comparing the pathogen count category to the pathogen compositional classes.

|  | Pathogen Compositional Class | | |
| --- | --- | --- | --- |
| Pathogen Count Category | Comp 1 | Comp2 | Comp 3 |
| 0 | 147 (25%) | 0 (0%) | 0 (0%) |
| 1 | 339 (55%) | 0 (0%) | 0 (0%) |
| 2-3 | 128 (20%) | 704 (77%) | 76 (73%) |
| 4+ | 0 (0%) | 286 (23%) | 28 (27%) |

**Supplemental Figure 1.** Weighted distribution of pathogens across different levels of pathogen burden counts, National Health and Nutrition Examination Survey (1999-2000).

**Supplemental Figure 2.** Distribution of log telomere length by HSV-1 (Panel A), HSV-2 (Panel B), CMV (Panel C), *H. pylori* (Panel D), Hepatitis B (Panel E), and pathogen burden composition class (Panel F).

A. HSV-1


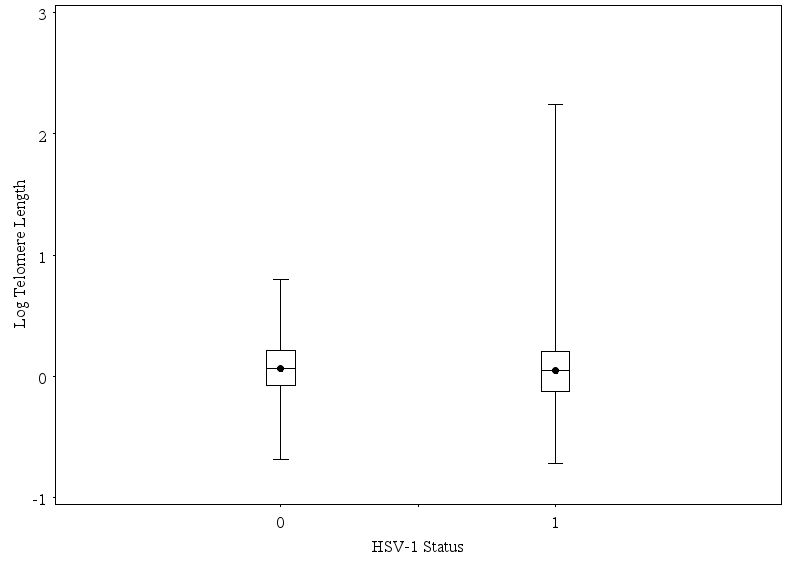


B. HSV-2


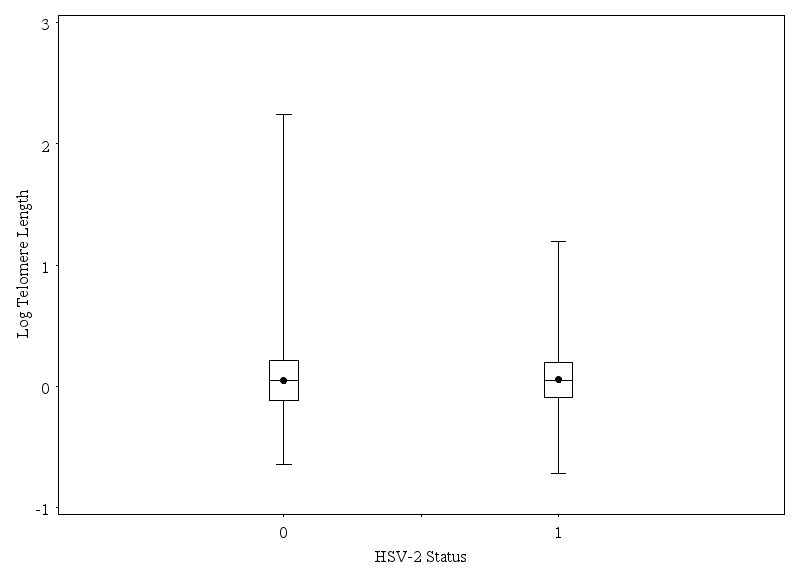


C. CMV


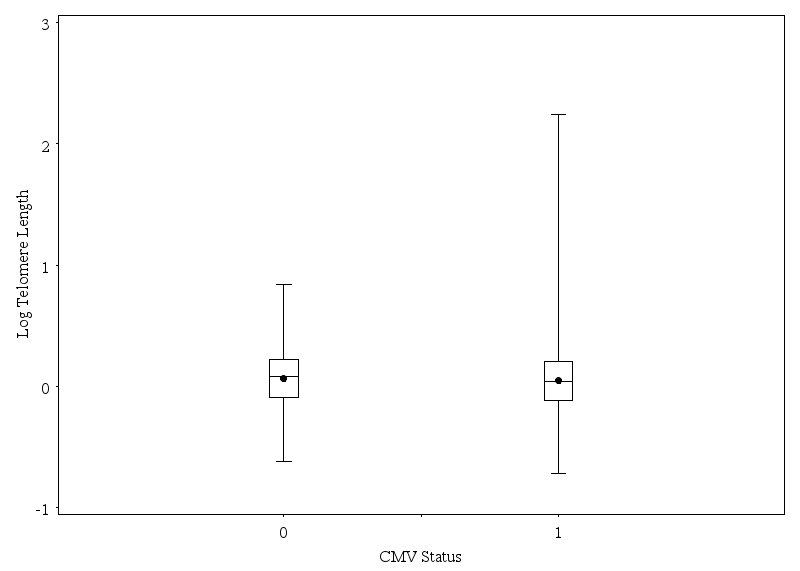


D. *H. pylori*


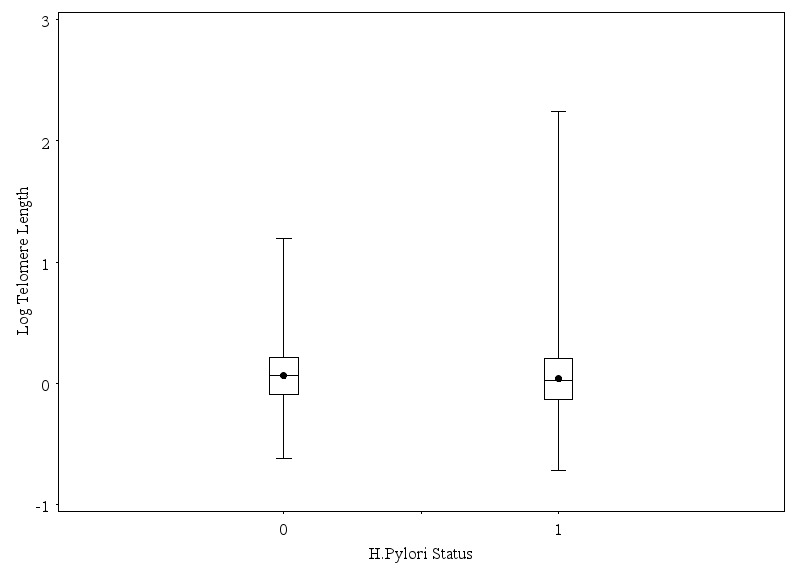


E. Hepatitis B


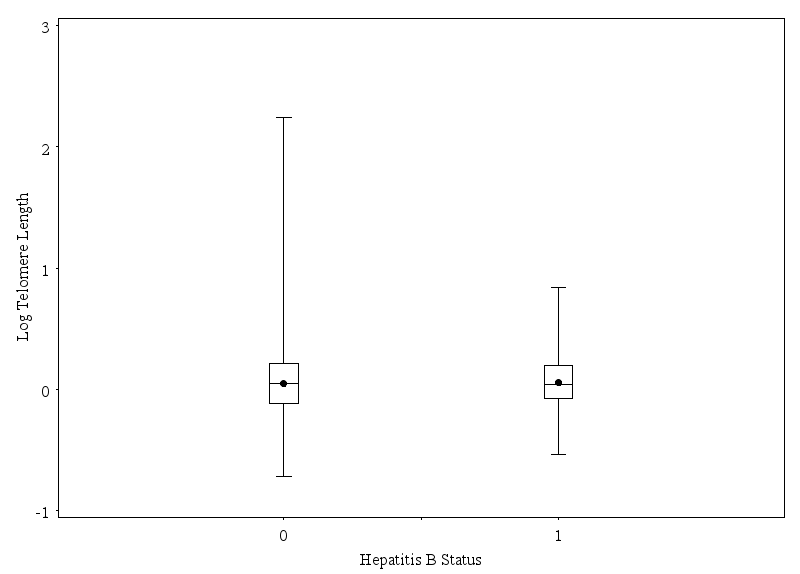


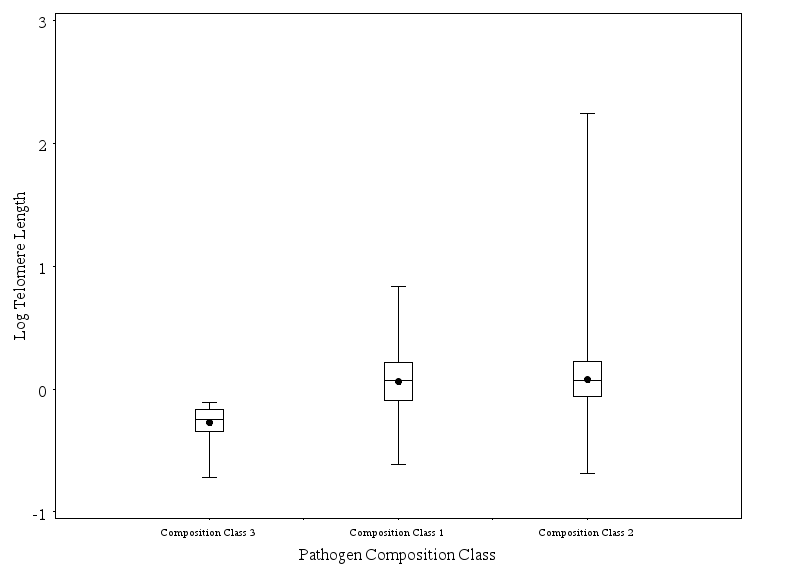
F. Pathogen Burden Composition Classes
